# Deep Learning-Based Privacy-Preserving Data Transmission Scheme for Clustered IIoT Environment

**DOI:** 10.1155/2022/8927830

**Published:** 2022-06-08

**Authors:** Kuruva Lakshmanna, R. Kavitha, B. T. Geetha, Ashok Kumar Nanda, Arun Radhakrishnan, Rachna Kohar

**Affiliations:** ^1^Department of Information Technology, Vellore Institute of Technology, Vellore, India; ^2^Department of CSE, Vel Tech Rangarajan Dr. Sagunthala R & D Institute of Science and Technology, Chennai, India; ^3^Department of ECE, Saveetha School of Engineering, SIMATS, Saveetha University, Chennai, India; ^4^CSE Department, B. V. Raju Institute of Technology, Narsapur, Medak, Telangana, India; ^5^Faculty of Electrical and Computer Engineering, Jimma Institute of Technology, Jimma University, Jimma, Ethiopia; ^6^School of Computer Science Engineering and Technology, Bennett University, Greater Noida, India

## Abstract

The Industrial Internet of Things (IIoT) has received significant attention from several leading industries like agriculture, mining, transport, energy, and healthcare. IIoT acts as a vital part of Industry 4.0 that mainly employs machine learning (ML) to investigate the interconnection and massive quantity of the IIoT data. As the data are generally saved at the cloud server, security and privacy of the collected data from numerous distributed and heterogeneous devices remain a challenging issue. This article develops a novel multi-agent system (MAS) with deep learning-based privacy preserving data transmission (BDL-PPDT) scheme for clustered IIoT environment. The goal of the BDL-PPDT technique is to accomplish secure data transmission in clustered IIoT environment. The BDL-PPDT technique involves a two-stage process. Initially, an enhanced moth swarm algorithm-based clustering (EMSA-C) technique is derived to choose a proper set of clusters in the IIoT system and construct clusters. Besides, multi-agent system is used to enable secure inter-cluster communication. Moreover, multi-head attention with bidirectional long short-term memory (MHA-BLSTM) model is applied for intrusion detection process. Furthermore, the hyperparameter tuning process of the MHA-BLSTM model can be carried out by the stochastic gradient descent with momentum (SGDM) model to improve the detection rate. For examining the promising performance of the BDL-PPDT technique, an extensive comparison study takes place and the results are assessed under varying measures. A significant amount of capital is required. It goes without saying that one of the most obvious industrial IoT concerns is the high cost of adoption. Secure data storage and management connectivity failures are common among IoT devices due to the massive amount of data they create. The simulation results demonstrate the enhanced outcomes of the BDL-PPDT technique over the recent methods. Despite the fact that the offered BDL-PPDT technique has an accuracy of just 98.15 percent, it produces the best feasible outcome. Because of the data analysis conducted as detailed above, it was determined that the BDL-PPDT technique outperformed the other current techniques on a range of different criteria and was thus recommended.

## 1. Introduction

Industrial Internet of Things (IIoT) employs actuators and sensors with communication and computation capabilities to transform the way the information is exchanged, analyzed, transformed, and collected into decisions [1]. This pervasive capability results in advanced Industry 4.0 (called Industrial Internet) application for enhanced efficiency and productivity in large industries like healthcare, energy, agriculture, mining, and transportation. The innovative Industry 4.0 features, namely, ML-based quality control and predictive maintenance and run-time reasoning, need to be simplified by distributed data acquisition [2]. In IIoT-based systems like open banking and smart healthcare, ML and data methods trained with the local boundary should be interacted with the branches or users to make organization-wider knowledge [3]. Often, vendors desired to limit their internal insight on product improvements and development with the organizational boundary for increasing business values against their contender. Furthermore, industries like open banking and smart healthcare are vastly convoluted with human-specific sensitive information [4]. ML model is trained on sensitive information that could expose confidential or private data to the attackers [5]. Therefore, trustworthiness and privacy are the key elements of ML in IIoT system. The Internet of Things is one of the primary drivers of the Industry 4.0 movement, since it enables greater automation, data collection, and analytics, as well as workflow and process optimization. The intelligence enabled by the Internet of Things enables devices to work cooperatively to produce outputs on an assembly line. MAS as a popular technique to offer trusts with distributed and decentralized settings might be employed in lots of potential applications, namely, supply chain management, IIoT, and healthcare [6]. The Internet of Things is a major driver of the Industry 4.0 movement since it enables increased automation, data collection, and analytics, as well as workflow and process optimization. The Internet of Things' intelligence enables devices to operate together on an assembly line to produce outputs. A multi-agent system (MAS or “self-organized system”) is a computerized system that is built of numerous intelligent agents that communicate with one another. Multi-agent systems are capable of resolving problems that a solo agent or a monolithic system would find difficult or impossible to solve. Methodical, functional, or procedural techniques, algorithmic search, or reinforcement learning can all be considered forms of intelligence. Among them, it is the mainstream application field, where blockchain is regarded as enabling technology for various applications. IIoT setup is a well-developed and also comprehensive deployment that can increase multiple challenges involving ensuring confidentiality, improving data accountability, availability, integrity, and availability (CIA). Blockchain can address this requirement and acts as a significant role by providing secure and verifiable solutions to store and share data [7]. IIoT application has requirements of similar kinds to guarantee trust and data integrity between several shareholders related to dissimilar parts of the logistic chain (e.g., storage, acquiring raw material, processing to customer, transportation, and industrial deployment). Also, in this application, requirements such as monitoring and maintaining history of each procedure are vital. The conventional security method has a number of constraints and does not fit for intelligent grid systems; for example, secured end-to-end encryption method could produce higher false alarm rate and interrupt analytical approach [8]. There is a considerable range of potential smart grid risks, like passive and active attacks. Another way of the attack is the smart grids, namely, sniffing the information from the CPS through open source data, and in active attack, the hackers can change the information through data poisoning attack or inference attack [9]. In data poisoning attacks, attackers attempt to change the standard information.

This article develops a novel MAS with deep learning-based privacy-preserving data transmission (BDL-PPDT) scheme for clustered IIoT environment. This research proposes a unique multi-agent system (MAS) method for clustered IIoT environments using deep learning-based privacy preserving data transmission (BDL-PPDT). The BDL-PPDT technique's objective is to achieve secure data transfer in a clustered IIoT environment. The BDL-PPDT technique involves the design of an enhanced moth swarm algorithm-based clustering (EMSA-C) technique for cluster head (CH) selection. In addition, blockchain technology (BCT) is applied for accomplishing secure inter-cluster communication. Furthermore, a new multi-head attention with bidirectional long short-term memory (MHA-BLSTM) model is used to find intrusions. To increase the detection rate, the stochastic gradient descent with momentum (SGDM) model can be used to tune the MHA-BLSTM model's hyperparameters. Finally, the stochastic gradient descent with momentum (SGDM)-based hyperparameter tuning process takes place. To inspect the significant performance of the BDL-PPDT technique, a wide-ranging comparative analysis is made and the results are inspected in terms of different measures.

## 2. Related Works

Sodhro et al. [10] proposed sustainable, secure, efficient, and reliable blockchain-driven methods. The presented method handles key arbitrarily by presenting the chain of blocks with a smaller amount of cores, less power drain, computation bit, and transmission. Next is an analytic hierarchy process (AHP) based smart decision-making method for the blockchaindriven that is more secured, reliable, sustainable, interoperable, and concurrent IIoT. Rahman et al. [11] presented a blockchain-based architecture to provision a verifiable query and privacy-preserving facilities to end-user in IIoT system. The architecture employs blockchain to save broad information as off-chain data and to save IoT information as on-chain data and provision search service to the user by performing a query in off-chain and on-chain data as well as generate an effective result.

Zhang et al. [12] developed a medical data privacy protection architecture-based blockchain (MPBC). In this method, they secure confidentiality by including different privacy noises to federated learning. Additionally, the increasing amount of healthcare data can make blockchain storage challenges. Thus, a storage mode is presented for reducing the storage burden of blockchain. The new information is locally stored and the hash values are estimated by IPFS and are saved in blockchain. Deebak and Al-Turjman [13] introduced a privacy-preserving smart contract with blockchain and artificial intelligence (PPSC-BCAI) architecture which facilitates system activities, human interaction, security risks, fraudulent claims, and service alerts. In order to examine the data sharing and transaction, an XGBoost is employed.

Weng et al. [14] proposed a secure, fair, and distributed DL architecture called DeepChain to resolve this problem. DeepChain provides a value-driven incentive method based on blockchain for forcing the participant to perform properly. In the meantime, DeepChain ensures data privacy for all the participants and provides auditability for the entire training procedure. Arachchige et al. [15] presented an architecture called PriModChain which forces trustworthiness and privacy on IIoT information by amalgamating federated ML, differential privacy, smart contracts, and Ethereum blockchain. The possibility of PriModChain based on resilience, privacy, security, reliability, and safety is estimated by the simulation technologically advanced in Python with socket programming on a multipurpose computer.

Kumar and Tripathi [16] designed a deep blockchain-based trustworthy privacy-preserving secured framework (DBTP2SF) for IIoT. This architecture contains two-phase privacy-preservation model, anomaly detection module, and trust management module. In the two-phase privacy model, a BC-enabled improved proof of work method is concurrently employed with AE, to convert cyber physical information to a novel form which avoids poisoning and inference attacks.

## 3. The Proposed Model

**Figure 1:**
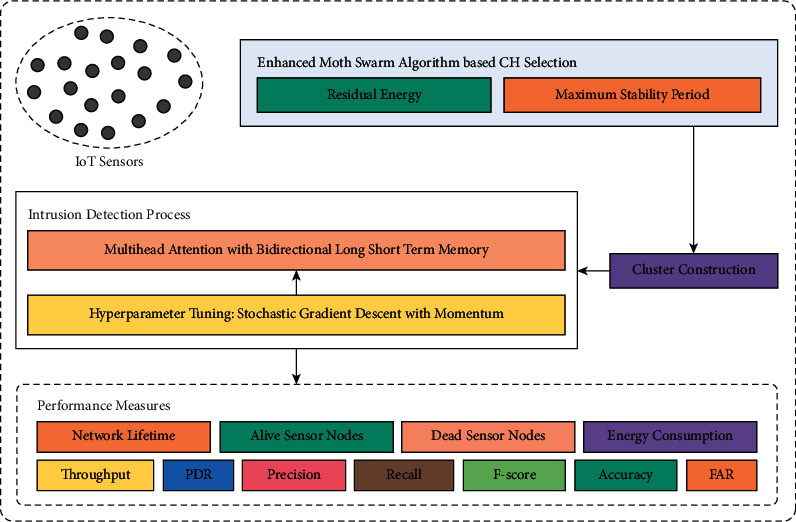
Working process of BDL-PPDT approach.

In this study, an effective BDL-PPDT technique has been developed to accomplish secure data transmission in clustered IIoT environment. The BDL-PPDT technique has presented a new EMSA-C technique to choose a proper set of clusters in the IIoT system and construct clusters. Next, the MHA-BLSTM with SGDM model is utilized for intrusion detection and the hyperparameter tuning process is made by the SGDM model resulting in improved detection performance.  Figure 1 illustrates the overall process of BDL-PPDT manner.

### 3.1. Process Involved in EMSA-C Technique

The nocturnal behaviors of moth are the motivation for the MSA [17]. In the model, the exploration and exploitation tradeoff considers a divider of candidate solutions generating the population:

- Onlooker (to exploit the optimal region discovered by the prospector).
- Pathfinder (to explore novel region of the searching space).
- Prospector (to exploit the novel regions attained by the pathfinder).

With other meta-heuristics models, this one begins with population initialization:

(1) $$\begin{array}{c} x_{ij}=r\text{ and}\cdot \left(u_{j}-l_{j}\right)+l_{j},\;\forall i\in \left\{,2,n\right\},\;j\in \left\{,2,d\right\}, \end{array}$$

whereas *u* and *l* represent maximum and minimum bounds of the searching space, *χ*_*i*_ denotes the candidate solution, *n* indicates the population size, *d* signifies the dimensionality of problems, and rand means an arbitrary number drawn from a uniform distribution. For generating pathfinder crossover, it is essential to estimate the variation coefficient and dispersal degree at iteration *t*:

(2) $$\begin{array}{c} \sigma_{j}^{t}=\frac{\sqrt{\left(1/n_{p}\right)\sum_{i=1}^{n_{p}}{\left(x_{ij}^{t}-P_{j}^{t}\right)}^{2}}}{P_{j}^{t}}, \end{array}$$

(3) $$\begin{array}{c} \mu^{t}=\frac{1}{d}\sum_{j=1}^{d}\sigma_{j}^{t}, \end{array}$$

whereas *n*_*p*_ represents the amount of pathfinders

(4) $$\begin{array}{c} P_{j}^{t}=\frac{1}{n_{p}}\sum_{i=1}^{n_{p}}xi_{ij}. \end{array}$$

In the MSA, the crossover point represents minimum dispersal value, as follows:

(5) $$\begin{array}{c} j\in c_{p}\text{ if }\sigma_{j}^{t}\leq \mu^{t}. \end{array}$$

Form this, *n*_*c*_ ∈ *c*_*p*_ crossover point is employed for creating a novel sub‐trial pathfinder vector $⟶\overset{⟶}{v_{p}}=\left[v_{p1},v_{p2},\ldots ,v_{pn_{c}}\right]$ from the novel pathfinder $\overset{⟶}{\chi_{p}}=\left[\chi_{p1},\chi_{p2},\ldots ,\chi_{pn_{c}}\right]$:

(6) $$\begin{array}{c} \overset{⟶}{v_{p}^{t}}=⟶x_{r^{1}}^{t}+L_{p1}^{t}\cdot \left(\overset{⟶}{x_{r^{2}}^{t}}-\overset{⟶}{x_{r^{3}}^{t}}\right)+L_{p2}^{t}\cdot \left(\overset{⟶}{x_{r^{4}}^{t}}-\overset{⟶}{x_{r^{5}}^{t}}\right), \end{array}$$

(7) $$\begin{array}{c} \forall r^{1}\neq r^{2}\neq r^{3}\neq r^{4}\neq r^{5}\neq p\in \left\{1,2,\ldots ,\;n_{p}\right\}. \end{array}$$

For each of the independent variables [18], the variables *L*_*pl*_ and *L*_*p*2_ are calculated using the Lévy stable distribution. There should only be one set of indexes *r* selected from the pathfinder solution, in which *L*_*pl*_ and *L*_*p*2_ represent independent variable calculated from the Lévy *α*‐stable distribution [18]. The set of indexes *r* should be only chosen from the pathfinder solution, and position is upgraded by the mutated variable extracted from the sub‐trail vector as follows:

(8) $$\begin{array}{c} V_{pj}^{t}=\left\{\begin{array}{cc} v_{pj}^{r} & \text{if }j\in c_{p},\\ x_{pj}^{t} & \text{if }j\notin c_{p}. \end{array}\right. \end{array}$$

Lastly, MSA employs a selection approach among the original and trial pathfinders as follows:

(9) $$\begin{array}{c} \overset{⟶}{x_{p}^{\text{t}+1}}=\left\{\begin{array}{c} \overset{⟶}{x_{p}^{\text{t}}}i\text{ if }f\left(\overset{⟶}{V_{p}^{t}}\right)\geq f\left(\overset{⟶}{x_{p}^{\text{t}}}\right),\\ \overset{⟶}{v_{p}^{t}}\text{ otherwise.} \end{array}\right. \end{array}$$

The possibility of choosing the next pathfinder is determined by

(10) $$\begin{array}{c} p_{p}=\frac{{\text{fit}}_{p}}{\sum_{p=1}^{np}{\text{fit}}_{p}}. \end{array}$$

That employs the luminescence intensity estimated as follows:

(11) $$\begin{array}{c} {\text{fit}}_{p}=\left\{\begin{array}{c} \frac{1}{1+f_{p}}\text{ if }f_{p}\geq 0,\\ 1+\left|f_{p}\right|\text{ otherwise.} \end{array}\right. \end{array}$$

From the pathfinder, *n*_*f*_ individual is chosen as prospector; this value is modified dynamically as follows:

(12) $$\begin{array}{c} n_{f}=\text{round}\left(\left(n-n_{p}\right)\times \left(1-\frac{t}{T}\right)\right), \end{array}$$

where *T* represents the maximal iteration number. The MSA enables the moth to move in a spiral manner over a pathfinder using equation (12):

(13) $$\begin{array}{c} \begin{array}{c} x_{i}^{t+1}=\left|x_{i}^{t}-x_{p}^{t}\right|\cdot e^{\theta }\cdot \text{cos}2\pi \theta +x_{p}^{t}\\ \forall p\in \left\{1,2,\ldots ,n_{p}\right\};\;i\in \left\{n_{p}+1,n_{p}+2,\ldots ,n_{f}\right\} \end{array}. \end{array}$$

Let *θ* ∈ [*r*, 1] be an arbitrary value employed for giving the spiral formation to the prospector path, while *r*=−1 − (*t*/*T*^·^).

The onlooker is the moth with the minimum luminescent intensity moving toward the shiniest source of light; in MSA, the onlooker is employed for intensifying the exploitation process. Further, the onlooker is separated into Gaussian walk and associative learning using immediate memory. Initially, the onlooker in the real iteration is attained as follows:

(14) $$\begin{array}{c} x_{i}^{\text{t}+1}=x_{i}^{t}+\varepsilon_{1}+\left[\varepsilon_{2}\cdot {}_{\text{best}}^{\text{gt}}-\varepsilon_{3}\cdot x_{i}^{t}\right],\;\forall i\in \left\{,2,\ldots ,n_{o}\right\}, \end{array}$$

whereas *ε*_2_ and *ε*_3_ represent uniformly distributed random value, *bes*t_g_ denotes the optimal candidate solution, *n*_*o*_=round(*n*_*u*_/2) indicates the amount of onlookers performing a Gaussian movement, *n*_*u*_ shows the amount of onlookers, and *ε*_1_ means an arbitrary value estimated by

(15) $$\begin{array}{c} \varepsilon_{1}∼\left.\text{random}\left(\text{size}\left(d\right)\right)⊕N\left({\text{best}}^{\text{t}}\right)\left(x_{i}^{t}-{\text{best}}_{g}^{t}\right)\right). \end{array}$$

The behavior of the moth considered short-term memory and associative learning is upgraded as follows:

(16) $$\begin{array}{c} \begin{array}{c} x_{i}^{t+1}=x_{i}^{t}+0.001\cdot G+\left(1-\frac{g}{G}\right)\cdot \varepsilon_{2}\cdot \left({\text{best}}_{p}-x_{i}^{t}\right)\\ +\left(\frac{2g}{G}\right)\cdot \varepsilon_{3}\cdot \left({\text{best}}_{p}^{t}-x_{i}^{t}\right),\;\forall i\in \left\{1,2,\ldots ,n_{m}\right\} \end{array}, \end{array}$$

with *n*_*m*_=*n*_*u*_ − *n*_*o*_ being the amount of onlookers performing short-term memory and associative learning; 1 − (*g*/*G*) indicates a cognitive factor, 2*g*/*G* represent a social factor, *besr*_*p*_ indicates the optimal light source from the pathfinder, and *G* ~ *N*(*x*_*i*_^*t*^ − *χ*_*i*_^min^, *χ*_*i*_^max^ − *χ*_*i*_^*t*^).

To improve the performance of the MSA, the EMSA is derived by the use of OBL concept. The efficient implementation of OBL contributes approximation of the opposite and current populations in the same generation for identifying optimum candidate solutions of a given problem. Object-based learning (OBL) is a student-centered learning approach that uses objects to facilitate deep learning. Objects may take many forms, small or large, but the method typically involves students handling or working at close quarters with and interrogating physical artefacts. The OBL models have been efficiently used in different meta-heuristics employed for improving convergence speed. The models of the opposite amount should be described in OBL.

Consider *N* ∈ *N*[*x*, *y*] to denote real numbers. The opposite numbers N0 are given by

(17) $$\begin{array}{c} N^{o}=x+y-N. \end{array}$$

In d-dimension searching region, the depiction may be extended as follows:

(18) $$\begin{array}{c} N_{i}^{o}=x_{i}+y_{i}-N_{i}, \end{array}$$

whereas (*N*_1_, *N*_2_,…*N*_*d*_) indicates d-dimension searching region and *N*_*i*_[*x*_*i*_, *y*_*i*_],  *i*=1,2,…, *d*. From the OBO, the approach of OBL is employed in this initiation procedure of MSA method and for all iterations in the application of jump rate.

Consider an IoT network of *n* sensor deployed arbitrarily. In order to be CH selective, the projected SSA executes squirrel population that was utilized by generating suitable clusters and maintaining the lower power employment of systems. Consider *X*=(*X*_1_, *X*_2_,…, *X*_*n*_) stands for the population vector of IoT with *n* sensors, where *X*_*i*_(*j*) ∈ {0,1}. The CH and normal nodes were signified as one and zero. The fundamental population of *NP* solution has inspired arbitrarily by representing 0 s as well as 1 s and representing as follows:

(19) $$\begin{array}{c} X_{i}\left(j\right)=\left\{\begin{array}{cc} 1, & \text{if}\left(r\text{ and }\leq p_{opt}\right),\\ 0, & \text{otherwise,} \end{array}\right. \end{array}$$

where *p*_*opt*_ stands for the recommended percentage of CHs and *r* and refers to uniform arbitrary values in zero and one. An arbitrarily located sensor node has been decided as *K* clusters: C_1_, C_2_,…, C_K_. The CH selective has responsible to decrease the cost of FF. Therefore, FF to CH selective was showcased as follows:

(20) $$\begin{array}{c} f_{{\text{obj}}_{-}\text{CH}}=\sum_{\text{i}=1}^{2}w_{i}\times f, \end{array}$$

with ∑_*i*=1_^2^*w*_*i*_=1. The maximum stability period is given by decreasing the Standard Deviation (SD) of the RE of node which is a significant issue. Therefore, SD (*σ*_*RE*_) is applicable to measure the control of uniformly distributed load in sensor node and illustrated as follows:

(21) $$\begin{array}{c} f_{1}=\sigma_{RE}\\ =\sqrt{\frac{1}{n}\sum_{j--1}^{n}{\left\{\mu_{RE}-E\left({\text{node}}_{\text{j}}\right)\right\}}^{2}}, \end{array}$$

where *μ*_*RE*_=(1/*n*)∑_*i*=1_^*n*^*E*(node_*i*_), *E*(node_*i*_) stands for the RE of *i*^*th*^ node, and *n* depicts the node count. A final objective was dependent upon clustering quality in that function of cluster isolation and cohesion has been implemented. Once the proportion of cohesion for separating was minimal, afterward optimum clustering was executed. It is accomplished by utilizing FF ratio of overall Euclidean distance of CH to CM and restricted Euclidean distance of 2 varying CHs.

(22) $$\begin{array}{c} f_{2}=Q_{c}\\ =\frac{\sum_{k=1}^{K}\sum_{\forall \;{\text{node}}_{j}\in C_{k}}d\left({\text{node}}_{j},CH_{k}\right)}{\;\min_{\forall C_{c},C_{k},C_{c}\neq C_{k}}\left\{d\left(CH_{c},CH_{k}\right)\right\}}. \end{array}$$

### 3.2. Secure Inter-Cluster Communication via Blockchain

**Figure 2:**
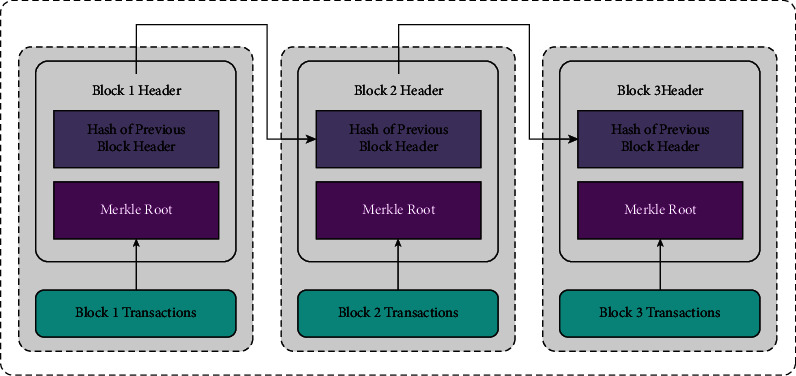
Structure of blockchain.

Generally, blockchain is assumed as a collection of blocks; also, a single block comprises of hash value of the existing block, information about the transaction (Ethereum, bitcoin), timestamp, and previous block. Furthermore, blockchain is determined as common and distributed digital ledger utilized to save the transaction data under different points. Therefore, when an attacker tries to derive information, it is not possible as every block has cryptographic value of the earlier block [19]. Now, each transaction is attained under the application of cryptographic hash values, viz. confirmed by all the miners. It consists of blocks of each transaction and captures same value of the comprehensive ledger.  Figure 2 illustrates the framework of blockchain. The blockchain offers the facility to share detailed ledgers in protective, confidential, and shared manner. Decentralized storage is the other source in blockchain, and the massive number of information data is linked and stored from existing blocks to earlier blocks through smart contract code. LitecoinDB, Swarm, SiacoinDB, MoneroDB, BigchainDB, Interplanetary File System (IPFS), and various factors were employed for decentralized dataset.

### 3.3. Intrusion Detection Process

During the intrusion detection process, the MHA-BLSTM with SGDM model is utilized. LSTM is a variant of RNN that could resolve gradient disappearance problems by presenting memory cell state, input gate *i*, output gate 0, and forget gate *f*. LSTM could enhance the memory model of NN for receiving training and input data that is appropriate to model time series data, such as text, owing to the design characteristics. BiLSTM is an integration of backward and forward LSTM. The greatest benefit of the model is that the sequence context data are taken fully into account. An LSTM unit contains controlling gate, along with IG *i*_*t*_, a forget gate *f*_*t*_, outcome gate 0_*t*_, and a memory cell state *c*_*t*_, that affects the unit capacity to update and store data. The IG outcome value lies between 0-1 according to the input *h*_*t*−1_ and *w*_*t*_. Once the outcome is 1, it implies that the cell state data are retained completely, and once the outcome is 0, it is abandoned completely. Then, the IG determines which value needs updating, and the  tanh  layer creates a novel candidate value vector $\tilde{c_{t}}$ that is added to the cell state. Next, both are integrated for updating the cell state *c*_*t*_; lastly, the outcome layer decides the outcome value based on the cell state. Among other, *W*_*f*_, *U*_*f*_, *b*_*f*_, *W*_*i*_, *U*_*i*_, *b*_*j*_, *W*_*c*_, *U*_*c*_, *b*_*c*_, and *W*_0_, *U*_0_, *b*_0_ represent the internal training parameter in the LSTM, *σ*(·) indicates sigmoid activation function, and ⊙ implies dot multiplication.

(23) $$\begin{array}{c} f_{t}=\sigma \left(W_{f}w_{t}+U_{f}h_{t-1}+b_{f}\right), \end{array}$$

(24) $$\begin{array}{c} i_{t}=\sigma \left(W_{i}w_{t}+U_{i}h_{t-1}+b_{i}\right), \end{array}$$

(25) $$\begin{array}{c} \tilde{c_{t}}=\tanh\left(W_{c}w_{c}+U_{c}h_{t-1}+b_{c}\right), \end{array}$$

(26) $$\begin{array}{c} c_{t}=i_{t}⊙\bar{c_{t}}+f_{t}⊙c_{t-1}, \end{array}$$

(27) $$\begin{array}{c} 0_{t}=\sigma \left(W_{0}w_{t}+U_{0}h_{t-1}+b_{0}\right), \end{array}$$

(28) $$\begin{array}{c} h_{t}=o_{t}\tanh\;⊙\left(c_{t}\right). \end{array}$$

The abovementioned method is the computation method of LSTM. As previously mentioned, BiLSTM comprises backward and forward LSTM. $\overset{⟶}{LSTM}$ in BiLSTM reads the input from *w*_1_ to *e*_*n*_ for generating $\overset{⟶}{h_{t}}$, and other $\overset{\leftarrow }{LSTM}$ reads the input from *e*_*n*_ to *w*_1_ for generating $\overset{\leftarrow }{h_{t-1}}$:

(29) $$\begin{array}{c} \overset{⟶}{h_{t}}=\overset{⟶}{LSTM}\left(w_{t},\overset{⟶}{h_{t-1}},c_{t-1}\right),\;t\in \left[1,m+n\right], \end{array}$$

(30) $$\begin{array}{c} \;\overset{\leftarrow }{h_{t}}=\overset{\leftarrow }{LSTM}\left(w_{t},\overset{\leftarrow }{h_{t-1}},c_{t-1}\right),\;t\in \left[m+n,1\right]. \end{array}$$

The reverse and forward context representations generated using $\overset{⟶}{h_{t}}$ and $\overset{\leftarrow }{h_{t}}$ are linked to the long vector,

(31) $$\begin{array}{c} h_{t}=\overset{⟶}{h_{t}}⊕\overset{\leftarrow }{h_{t}}. \end{array}$$

Lastly, the output [*h*_1_,…*h*_*i*_,…*h*_*m*_, *l*_1_,…*l*_*j*_ … *l*_*n*_] of the entire sentence is attained, whereas *h*_*i*_ and *l*_*j*_ are exploited to signify the output of emoticons and words, correspondingly. Furthermore, set each intermediate layer in BiLSTM for returning the comprehensive output sequence, thus ensuring that the output of all the hidden layers retains the longer‐distance data as possible.

Attention mechanism is used to improve the effects of RNN-based model, and also it consists of dot-product attention and additive attention [20]. The calculation of attention is separated into 3 stages. Initially, utilize F attention function to score key and query to get si; next, utilize softmax function to standardize the scoring results si, for obtaining the weight ai. Lastly, estimate attention that is the weighted average of each value and weight ai. Multi-head attention mechanism has enhanced the classical attention method; thus, all the heads could extract the features of key and query in distinct subsets. More precisely, this feature comes from *Q* and *K* that is the projection of key and query in the subspaces. Note that in the multi-head attention model, the attention functions can be the scaled dot-product function that is similar to the classical attention mechanism, excepting the regulating scaling factors [21–27]. In this work, *h* should be debugged continuously for determining the appropriate values. Lastly, the result, i.e., returned in every head, is linearly converted and concatenated to attain multi-head attention. Eventually, transmit the vector from the preceding layer to the densely connected layer. They utilize ReLU function as the activation function for completing the nonlinear transformation. Finally, execute the softmax function on the output of the preceding layer and attain intrusion detection output.

For optimally adjusting the hyperparameter of the MHA-BLSTM model, the SGDM is applied. SGDM is a first-order momentum depending on SGD. The 1st-order momentum represents the exponential moving of the gradient direction at all the moments, nearly equivalent to sum of the gradient vector at the current *T*_*j*_ moment. And, the *T*_*j*_ is denoted by

(32) $$\begin{array}{c} T_{j}=\frac{1}{1-\beta_{i}}. \end{array}$$

In another word, the descendant direction at *t* time is described using the descending direction accumulated before as well as gradient direction of the existing point. The empirical value of *β*_1_ is 0.9, which implies the direction of decline is particularly the before accumulated direction of decline.

## 4. Results and Discussion

**Figure 3:**
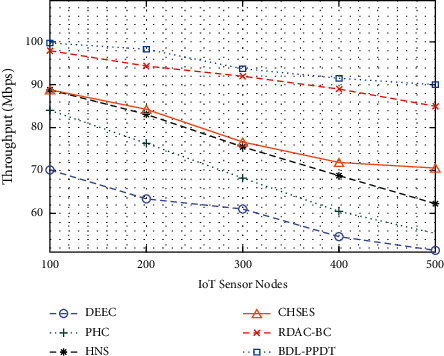
Throughput analysis of BDL-PPDT technique with existing approaches.

In this section, a detailed experimental validation of the BDL-PPDT technique takes place under varying numbers of IoT sensor nodes and rounds. The results are examined in varying aspects. An extensive throughput analysis of the BDL-PPDT technique with other methods is given in Table 1 and Figure 3. The results reported that the BDL-PPDT technique has demonstrated enhanced throughput under every IoT sensor node. For instance, with 100 IoT sensor nodes, the BDL-PPDT technique has offered improved throughput of 99.71 Mbps whereas the DEEC, PHC, HNS, CHSES, and RDAC-BC techniques have accomplished decreased NSAN of 69.98 Mbps, 84.17 Mbps, 88.89 Mbps, 88.68 Mbps, and 98.16 Mbps, respectively. Moreover, with 500 IoT sensor nodes, the BDL-PPDT technique has accomplished raised throughput of 89.72 Mbps, whereas the DEEC, PHC, HNS, CHSES, and RDAC-BC techniques have led to lessening NSAN of 51.48 Mbps, 55.31 Mbps, 62.24 Mbps, 70.42 Mbps, and 85.05 Mbps, respectively.

**Figure 4:**
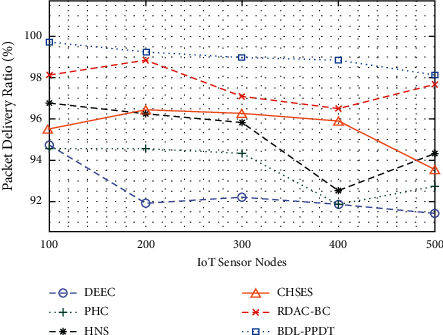
PDR analysis of BDL-PPDT technique with existing approaches.

Figure 4 offers the detailed PDR analysis of the BDL-PPDT technique under several IoT sensor nodes. From the results, it can be observed that the BDL-PPDT technique has reported enhanced PDR under every IoT sensor node. For instance, with 100 IoT sensor nodes, the BDL-PPDT technique has gained an increased PDR of 99.72%, whereas the DEEC, PHC, HNS, CHSES, and RDAC-BC techniques have reached to decrease PDR of 94.74%, 94.57%, 96.80%, 95.48%, and 98.11%, respectively. Besides 500 IoT sensor nodes, the BDL-PPDT technique has exhibited a maximum PDR of 98.16%, whereas the DEEC, PHC, HNS, CHSES, and RDAC-BC techniques have depicted minimum PDR of 91.45%, 92.76%, 94.39%, 93.53%, and 97.69%, respectively.

**Figure 5:**
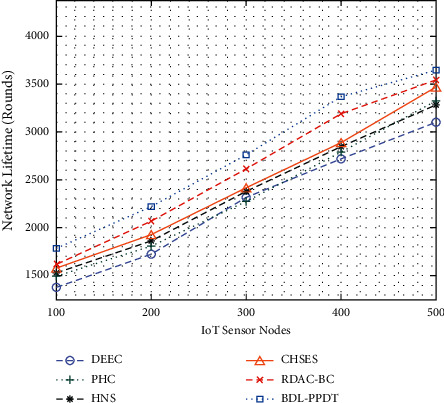
NLT analysis of BDL-PPDT technique with existing approaches.

A brief comparative NLT analysis of the BDL-PPDT technique is illustrated in Table 2 and Figure 5. From the results, it is evident that the BDL-PPDT technique has provided supreme NLT under every IoT sensor node. For instance, with 100 IoT sensor nodes, the BDL-PPDT technique has given superior NLT of 1793 rounds, whereas the DEEC, PHC, HNS, CHSES, and RDAC-BC techniques have offered inferior NLT of 1386, 1492, 1529, 1588, and 1612 rounds, respectively. Eventually, with 500 IoT sensor nodes, the BDL-PPDT technique has exhibited higher NLT of 3633 rounds, whereas the DEEC, PHC, HNS, CHSES, and RDAC-BC techniques have attained lower NLT of 3103, 3326, 3289, 3463, and 3547 rounds, respectively.

**Figure 6:**
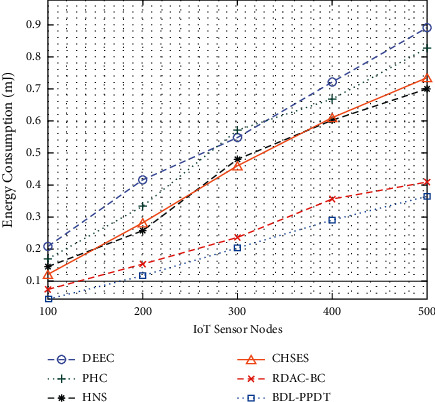
ECM analysis of BDL-PPDT technique with existing approaches.

The ECM analysis of the BDL-PPDT technique with other methods under distinct IoT sensor nodes is represented in Figure 6. The results inferred that the BDL-PPDT technique has managed to offer minimal ECM under all IoT sensor nodes. For instance, with 100 IoT sensor nodes, the BDL-PPDT technique has achieved minimal ECM of 0.0470 mJ, whereas the DEEC, PHC, HNS, CHSES, and RDAC-BC techniques have obtained maximum ECM of 0.2058 mJ, 0.1690 mJ, 0.1425 mJ, 0.1165 mJ, and 0.0756 mJ, respectively. Furthermore, with 500 IoT sensor nodes, the BDL-PPDT technique has offered a least ECM of 0.3654 mJ, whereas the DEEC, PHC, HNS, CHSES, and RDAC-BC techniques have reached to an increased ECM of 0.8872 mJ, 0.8277 mJ, 0.7007 mJ, 0.7351 mJ, and 0.4084 mJ, respectively.

**Figure 7:**
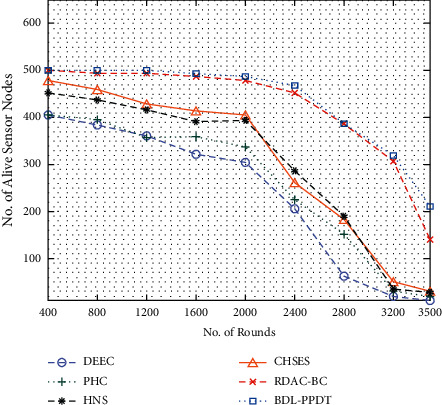
NASN analysis of BDL-PPDT technique with varying rounds.

A brief comparative number of alive sensor node (NASN) analysis of the BDL-PPDT technique takes place in Table 3 and Figure 7. From the results, it can be noticed that the BDL-PPDT technique has accomplished maximum NASN under every round. For instance, with 800 rounds, the BDL-PPDT technique has provided higher NASN of 499, whereas the DEEC, PHC, HNS, CHSES, and RDAC-BC techniques have gained lower NSAN of 384, 394, 436, 458, and 495 nodes, respectively. Besides, with 3500 rounds, the BDL-PPDT technique has resulted in improved NASN of 210, whereas the DEEC, PHC, HNS, CHSES, and RDAC-BC techniques have led to reduced NSAN of 12, 19, 28, 30, and 138 nodes, respectively.

**Figure 8:**
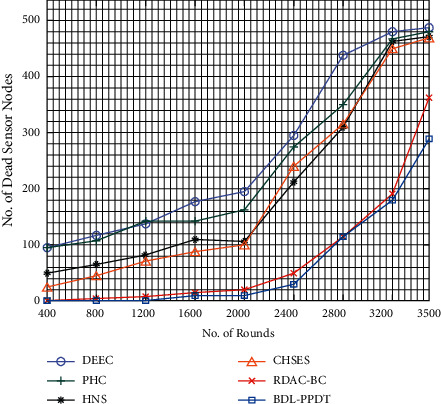
NDSN analysis of BDL-PPDT technique with varying rounds.

The number of dead sensor node (NDSN) analysis of the BDL-PPDT technique with other methods under distinct rounds is given in Table 4 and Figure 8. The results implied that the BDL-PPDT technique has attained effective outcomes with the lower NDSN under all rounds. For instance, with 800 rounds, the BDL-PPDT technique has achieved minimal NDSN of 1, whereas the DEEC, PHC, HNS, CHSES, and RDAC-BC techniques have obtained maximum NDSN of 116, 106, 64, 42, and 5 nodes, respectively [28–31]. At the same time, with 3500 rounds, the BDL-PPDT technique has offered a least NDSN of 290, whereas the DEEC, PHC, HNS, CHSES, and RDAC-BC techniques have reached to an increased NDSN of 488, 481, 472, 470, and 362 nodes, respectively.

**Figure 9:**
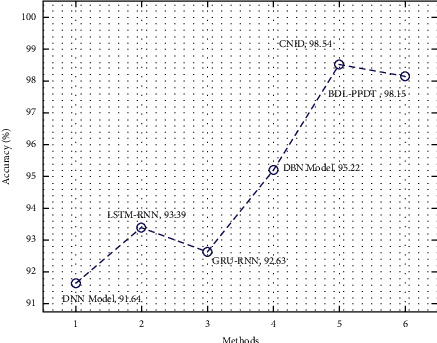
Accuracy analysis of BDL-PPDT technique with existing approaches.

Here, the intrusion detection performance analysis of the BDL-PPDT technique is provided in Table 5 and Figure 9 [21, 22]. The results are tested using the KDDCup99 dataset [23] comprising different classes and 41 features. The results show that the DNN model has gained lower outcomes with the *accu*_*y*_ of 91.64%, whereas the LSTM-RNN and GRU-RNN techniques have resulted in moderately reasonable *accu*_*y*_ of 93.39% and 92.63%, respectively [35–38]. Moreover, the DBN and CNID models have accomplished considerable *accu*_*y*_ values of 95.22% and 98.54%, respectively. However, the presented BDL-PPDT technique has reached to maximum outcome with the *accu*_*y*_ of 98.15%. The abovementioned result analysis implied that the BDL-PPDT technique has outperformed the other existing techniques in terms of different measures.

## 5. Conclusion

In this study, an effective BDL-PPDT technique has been developed to accomplish secure data transmission in clustered IIoT environment. The BDL-PPDT technique has presented a new EMSA-C technique to choose a proper set of clusters in the IIoT system and construct clusters. Next, the MHA-BLSTM with SGDM model is utilized for intrusion detection and the hyperparameter tuning process is made by the SGDM model resulting in improved detection performance. To inspect the significant performance of the BDL-PPDT technique, a wide-ranging comparative analysis is made and the results are inspected in terms of different measures. The experimental outcome pointed out the improved performance of the BDL-PPDT technique over the recent methods in terms of different measures. In the future, hyperparameter tuning process of the MHA-BLSTM model can be done by the meta-heuristic algorithms to improve the overall performance. Even though the BDL-PPDT method has an accuracy rate of just 98.15 percent, it still gives the best possible result. Because of the data analysis above, it was found that the BDL-PPDT technique outperformed the other current techniques on a wide range of different factors, and so it was recommended that people use it. Meta-heuristic methods will be utilized in the future to modify the hyperparameters of the MHA-BLSTM model, resulting in an overall improvement in overall performance.

## Figures and Tables

**Table 1:** Result analysis of BDL-PPDT technique with existing approaches.

Packet delivery ratio (%)						
IoT sensor nodes	DEEC	PHC	HNS	CHSES	RDAC-BC	BDL-PPDT
100	94.74	94.57	96.80	95.48	98.11	99.72
200	91.93	94.61	96.26	96.44	98.87	99.23
300	92.21	94.32	95.84	96.22	97.10	98.97
400	91.86	91.89	92.53	95.88	96.50	98.84
500	91.45	92.76	94.39	93.53	97.69	98.16

Throughput (Mbps)						
IoT sensor nodes	DEEC	PHC	HNS	CHSES	RDAC-BC	BDL-PPDT

100	69.98	84.17	88.89	88.68	98.16	99.71
200	63.40	76.46	83.26	84.32	94.27	98.42
300	61.05	68.33	75.50	76.67	92.03	93.80
400	54.68	60.53	68.89	71.76	88.97	91.57
500	51.48	55.31	62.24	70.42	85.05	89.72

**Table 2:** Comparative analysis of BDL-PPDT technique with varying IoT sensor nodes.

Energy consumption (mJ)						
IoT sensor nodes	DEEC	PHC	HNS	CHSES	RDAC-BC	BDL-PPDT
100	0.2058	0.1690	0.1425	0.1165	0.0756	0.0470
200	0.4164	0.3315	0.2576	0.2761	0.1496	0.1176
300	0.5478	0.5684	0.4784	0.4635	0.2343	0.2017
400	0.7226	0.6687	0.6027	0.6048	0.3570	0.2872
500	0.8872	0.8277	0.7007	0.7351	0.4084	0.3654

Network lifetime (rounds)						
IoT sensor nodes	DEEC	PHC	HNS	CHSES	RDAC-BC	BDL-PPDT
100	1386	1492	1529	1588	1612	1793
200	1725	1807	1864	1918	2077	2218
300	2305	2271	2389	2405	2613	2756
400	2718	2789	2853	2885	3191	3362
500	3103	3326	3289	3463	3547	3633

**Table 3:** NASN analysis of the BDL-PPDT technique with different rounds.

No. of alive sensor nodes						
No. of rounds	DEEC	PHC	HNS	CHSES	RDAC-BC	BDL-PPDT
400	404	406	451	476	500	500
800	384	394	436	458	495	499
1200	361	357	418	427	492	497
1600	322	359	390	412	486	492
2000	304	338	395	403	479	489
2400	205	227	288	259	451	470
2800	62	151	190	184	386	387
3200	20	32	37	50	309	320
3500	12	19	28	30	138	210

**Table 4:** NDSN analysis of the BDL-PPDT technique with different rounds.

No. of dead sensor nodes						
No. of rounds	DEEC	PHC	HNS	CHSES	RDAC-BC	BDL-PPDT
400	96	94	49	24	0	0
800	116	106	64	42	5	1
1200	139	143	82	73	8	3
1600	178	141	110	88	14	8
2000	196	162	105	97	21	11
2400	295	273	212	241	49	30
2800	438	349	310	316	114	113
3200	480	468	463	450	191	180
3500	488	481	472	470	362	290

**Table 5:** Comparative analysis of BDL-PPDT technique with different measures.

Methods	Accuracy	Precision	Recall	F1-score	Far
DNN model	91.64	97.85	91.99	94.67	8.56
LSTM-RNN	93.39	98.11	94.41	96.12	6.81
GRU-RNN	92.63	97.52	93.45	95.34	7.57
DBN model	95.22	97.55	96.50	97.11	3.98
CNID	98.54	99.98	97.56	98.49	0.02
BDL-PPDT	98.15	99.99	98.64	98.96	0.01

## Data Availability

The article contains all of the data.
